# Editorial: Wildlife-domestic animal interface: threat or sentinel?

**DOI:** 10.3389/fvets.2024.1495580

**Published:** 2024-09-24

**Authors:** Gianmarco Ferrara, Carlos Tejeda

**Affiliations:** ^1^Department of Veterinary Medicine and Animal Production, University of Naples, Federico II, Naples, Italy; ^2^Universidad Austral de Chile, Facultad de Ciencias Veterinarias, Departamento de Medicina Preventiva, Valdivia, Chile

**Keywords:** wildlife, sentinel, threat, pathogens, wildlife-livestock transmission, wildlife-human interactions

## 1 Introduction

In general, wildlife can serve as reservoirs and sentinels for a range of transmissible diseases (1). Furthermore, wildlife may be exposed to domestic animal diseases, which can have major consequences for their populations (2). Wildlife monitoring and surveillance, particularly from a health point of view, is a critical requirement for major infection decrease and an essential component of conservation and management initiatives (3).

## 2 Wildlife vs. pets

Wildlife may be a major source of infection for pets. Rabies, pseudorabies, *Leptospira* spp., distemper, and canine viral gastroenteritis are classic examples of pathogens that can take advantage of interactions between these two animal species (4, 5). Interactions between the domestic/wild entities may result in epidemics in both populations, the emergence of novel strains or variants, and spillover events (6). Scientific literature is abundant in studies of prevalence and seroprevalence of pathogens in wild animals transmissible to domestic animals (5, 7). Often, these studies include several mammal species. An outbreak of feline parvovirus (FPV-2) in Pallas' cats in a wildlife park in China was recently described by Wei et al., who succeeded in virus isolation and characterization. This epidemic emphasizes the critical necessity for continuous epidemiological surveillance and severe disinfection measures to avoid FPV spread in wildlife parks.

## 3 Wildlife vs. livestock

The interaction between wildlife and livestock has always been challenging due to the huge range of infections that may be transmitted (6). Wild ruminants and wild boars may carry bacteria and viruses that are under eradication plans at the domestic interface, posing issues owing to the damage that these diseases bring to animal production (8–11). The most recent and famous example is African swine fever, but other infections use similar dynamics to spread, causing less apparent but no less significant damage (12). Recently, a systematic review written by Dagnaw et al., established that the global prevalence of exposure to Schmallenberg virus (SBV), an impactful peribunyavirus of ruminants, is 49% in domestic ruminants and 26% in wild ones (red deer, roe deer, fallow deer, and mouflon) (13). According to the subgroup analysis, cattle had the greatest pooled prevalence of SBV (59%), followed by sheep (37%), and goats (18%). The sub-pooled incidence of SBV was highest in roe deer (46%), followed by fallow deer (30%), red deer (27%), mouflon (22%), and wild boar (11%). Other evidence has reported the presence of *Mycoplasma bovis* in alpine chamois (*Rupicapra rupicapra*) in Italy (Bullone et al.) and the exposure to *Toxoplasma gondii, Neospora caninum, Coxiella burnetii, Brucella* spp., *Chlamydophila abortus, Mycobacterium avium* subsp. *paratuberculosis* (MAP), and *Mycobacterium bovis* in wild ruminants in Slovenia (Žele Vengušt et al.).

## 4 Wildlife vs. humans

The incidence of wildlife-human encounters has increased due to continuous urbanization and the loss of wild animal habitats (14). One of the most devastating effects of a pathogen's existence in a natural population is the spread of infection to people. The potential spread of SARS-CoV-2 from wild animals to humans was probably responsible for one of the greatest pandemics ever recorded (15, 16). The presence of the influenza virus in wildlife is very dangerous (17). Alava et al., reflects on the presence of this virus in pinnipeds of the Galápagos Islands. However, wildlife can also transmit the bacteria to humans. For example, Mateus-Vargas et al. have described that American crocodiles (*Crocodylus acutus*) in Costa Rica carry tetracycline-resistant *Escherichia coli*. Extended-spectrum β-lactamase (ESBL)-producing *Escherichia coli* (ESBL-EC) with high diversity of resistance and virulence elements have been reported by Liu et al. in giant pandas. These factors have major consequences for the design of environmental monitoring programs employing such specimens. When considering human-crocodile and human-panda conflicts from a One Health viewpoint, the emergence of antimicrobial resistance highlights the significance of rigorous monitoring of antibiotic resistance development in wildlife.

## 5 Wildlife and conservation

Pathogens have a substantial influence on animal populations, resulting in the loss of biodiversity and ecological services. A key aspect of wildlife conservation is knowledge. Numerous studies have studied the microbiota of the most disparate species, which helps to understand the composition of the intestinal bacterial flora and therefore of eating habits, the colonization by zoonosis bacterial etc. (18). Recently, Wang et al., have analyzed the composition and functional structures of the gut microbiota of Himalayan griffons under wild and captive conditions, finding no significant differences in the alpha diversity between the two groups, but significant differences in beta diversity. This work is an important initial step to a larger investigation of scavenger microbiomes, with the eventual objective of contributing to conservation and management methods for this near-threatened species. These metagenomic approaches are useful in providing new insights into the microbiome and virome of wild species.

## 6 In summary

Domestic animal-wildlife interaction is a growing global concern. Throughout history, wildlife has been a major source of infection transmissible to domestic animals, and when this transmission includes zoonoses, it becomes a serious public health concern affecting all continents. According to the most recent scientific findings, surveillance and monitoring are critical for completely understanding the magnitude of disease dissemination and preventing spillover to domestic animals and humans.
